# *LINC00152* Drives a Competing Endogenous RNA Network in Human Hepatocellular Carcinoma

**DOI:** 10.3390/cells11091528

**Published:** 2022-05-03

**Authors:** Rossella Pellegrino, Mirco Castoldi, Fabio Ticconi, Britta Skawran, Jan Budczies, Fabian Rose, Constantin Schwab, Kai Breuhahn, Ulf P. Neumann, Nadine T. Gaisa, Sven H. Loosen, Tom Luedde, Ivan G. Costa, Thomas Longerich

**Affiliations:** 1 Institute of Pathology, Heidelberg University Hospital, 69120 Heidelberg, Germany; jan.budczies@med.uni-heidelberg.de (J.B.); fabian.rose@med.uni-heidelberg.de (F.R.); constantin.schwab@med.uni-heidelberg.de (C.S.); kai.breuhahn@med.uni-heidelberg.de (K.B.); thomas.longerich@med.uni-heidelberg.de (T.L.); 2 Clinic for Gastroenterology, Hepatology and Infectious Diseases, University Hospital Düsseldorf, Medical Faculty of Heinrich Heine University Düsseldorf, 40225 Düsseldorf, Germany; mirco.castoldi@med.uni-duesseldorf.de (M.C.); sven.loosen@med.uni-duesseldorf.de (S.H.L.); tom.luedde@med.uni-duesseldorf.de (T.L.); 3 Institute for Computational Genomics, Joint Research Center for Computational Biomedicine, University Hospital RWTH Aachen, 52074 Aachen, Germany; fabio.ticconi@gmail.com (F.T.); ivan.costa@rwth-aachen.de (I.G.C.); 4 Institute of Human Genetics, Hannover Medical School, 30625 Hannover, Germany; skawran.britta@mh-hannover.de; 5 Department of General, Visceral and Transplant Surgery, University Hospital RWTH Aachen, 52074 Aachen, Germany; uneumann@ukaachen.de; 6 Department of Surgery, Maastricht University Medical Centre, 6229 HX Maastricht, The Netherlands; 7 Institute of Pathology, University Hospital RWTH Aachen, 52074 Aachen, Germany; ngaisa@ukaachen.de

**Keywords:** HCC, cell proliferation, promoter methylation, ceRNA network, KLC2, miR-143a-3p, *LINC00152*

## Abstract

Genomic and epigenomic studies revealed dysregulation of long non-coding RNAs in many cancer entities, including liver cancer. We identified an epigenetic mechanism leading to upregulation of the long intergenic non-coding RNA 152 (*LINC00152*) expression in human hepatocellular carcinoma (HCC). Here, we aimed to characterize a potential competing endogenous RNA (ceRNA) network, in which *LINC00152* exerts oncogenic functions by sponging miRNAs, thereby affecting their target gene expression. Database and gene expression data of human HCC were integrated to develop a potential *LINC00152*-driven ceRNA in silico. RNA immunoprecipitation and luciferase assay were used to identify miRNA binding to *LINC00152* in human HCC cells. Functionally active players in the ceRNA network were analyzed using gene editing, siRNA or miRNA mimic transfection, and expression vectors in vitro. RNA expression in human HCC in vivo was validated by RNA in situ hybridization. Let-7c-5p, miR-23a-3p, miR-125a-5p, miR-125b-5p, miR-143a-3p, miR-193-3p, and miR-195-5p were detected as new components of the potential *LINC00152* ceRNA network in human HCC. *LINC00152* was confirmed to sponge miR143a-3p in human HCC cell lines, thereby limiting its binding to their respective target genes, like *KLC2*. KLC2 was identified as a central mediator promoting pro-tumorigenic effects of *LINC00152* overexpression in HCC cells. Furthermore, co-expression of *LINC00152* and *KLC2* was observed in human HCC cohorts and high *KLC2* expression was associated with shorter patient survival. Functional assays demonstrated that KLC2 promoted cell proliferation, clonogenicity and migration in vitro. The *LINC00152*-miR-143a-3p-KLC2 axis may represent a therapeutic target in human HCC.

## 1. Introduction

During the last two decades comprehensive genomic and epigenomic studies resulted in molecular classification proposals of human HCC [1,2,3,4]. Of note, most of the human transcriptome represent non-protein coding genes [5,6]. A large group of these non-coding transcripts are long non-coding RNAs (lnc-RNAs), which are, by definition, composed of more than 200 nucleotides [5]. Several studies demonstrated that lnc-RNAs regulate gene and protein expression, thereby playing a key role in several cellular processes like embryonic development, but also in the pathogenesis of diseases, in particular tumorigenesis [7]. Consequently, altered lnc-RNA expression was detected in many cancer entities, including HCC [8,9]. By integration of gene expression and promoter methylation we have described that *LINC00152* (also known as *CYTOR*), an intergenic lnc-RNA, is significantly upregulated in human HCC as a consequence of promoter hypomethylation [10]. Interestingly, *LINC00152* expression was further detected dysregulated in other human cancer entities, such as gastric, colon, gallbladder and renal carcinomas, in which its upregulation promotes tumor cell proliferation, migration, invasion, epithelial to mesenchymal transition, and chemoresistance [11]. Furthermore, *LINC00152* was shown to promote proliferation, migration and invasiveness of liver cancer cells via various mechanisms, including transcriptional dysregulation [12] and altered miRNA binding [13,14].

*LINC00152* has been proposed to act as a competing endogenous RNA (ceRNA) in gastric cancer [15]. Conceptually, lncRNAs are able to bind miRNAs through homologous miRNA responsive elements (MRE), thereby regulating miRNA bioavailability. As a consequence, mRNAs sharing MREs may be regulated by a common lnc-RNA [16].

Based on in silico prediction, we determined dysregulated *LINC00152*-binding miRNA target genes leveraging genome-wide gene expression data of human HCC. This comprehensive approach facilitated the identification of a *LINC00152*-driven ceRNA network in human HCC, in which the limited bioavailability of miR-143a-3p leads to upregulation of Kinesin Light Chain 2 (KLC2) and consequently increased tumor cell proliferation and migration.

## 2. Materials and Methods

### 2.1. Gene and miRNA Expression Profiling

We used 41 human HCCs, derived from 38 patients, to generate gene expression profiles, as described previously [10] and which are publicly available as GEO dataset (GSE50579). Human tissue samples and clinical data were provided by the Tissue Bank of the National Center for Tumor Diseases Heidelberg. All diagnoses were confirmed by histological re-evaluation and use of the samples was approved by the local ethics committee (S206-05) in compliance with the Helsinki Declaration. According to the vote, informed consent was not required because only long-term archived (>5 years), pseudonymized samples were used for this study. Cohort characteristics are shown in Table S1. For the analysis of the TCGA HCC cohort (corresponding to the LIHC cohort) [4], expression profiling and clinical data were downloaded from the PanCanAtlas website (https://gdc.cancer.gov/about-data/publications/pancanatlas, accessed on 12 May 2021) [17].

### 2.2. Construction and Modeling of the *LINC00152*-Driven ceRNA Network

The miRcode database (http://www.mircode.org, accessed on 18 December 2014) was used to predict miRNAs able to bind to the *LINC00152* sequence (Table S2). For prediction of corresponding miRNA target genes miRecords (http://c1.accurascience.com/miRecords/, accessed on 18 December 2014), TarBase 7.0 (http://diana.imis.athena-innovation.gr/DianaTools/index.php?r=tarbase/index, accessed on 18 December 2014), and starBase V2.0 (https://starbase.sysu.edu.cn/starbase2/, accessed on 18 December 2014) databases were exploited. Potential target genes predicted by at least two of these algorithms (*n* = 204) were selected for further analysis with R software. The association between each gene in the dataset GSE50579 and *LINC00152* expression, across the whole cohort, was assessed via Pearson correlation. Further, the relevance of each predicted miRNA was evaluated by comparing the absolute *LINC00152*-correlation of its target genes with the absolute *LINC00152*-correlation of all potential target genes as background (Welch *t*-test, *p* < 0.05 and adjusted via Benjamini-Hochberg procedure).

### 2.3. RNA In Situ Hybridization Using Tissue Microarray

Human tissue samples for construction of a tissue microarray (TMA) were provided by the Institute of Pathology, University Hospital Aachen. All diagnoses were confirmed by histological re-evaluation and use of the samples was approved by the local ethics committee (EK122/16) in compliance with the Helsinki Declaration. According to the vote, informed consent was not required because only long-term archived (>5 years) anonymized samples were used. The TMA contained tissue from non-tumorous liver tissue of HCC patients (*n* = 42), and HCCs (*n* = 50; Table S3) and was constructed as previously described [18], and RNA in situ hybridization using the RNAscope technology (Advanced Cell Diagnostics, Newark, CA, USA) was performed on 5 µm sections. Tissue slides were stained by following the manufacturer’s instructions for RNAscope 2.5 HD-Red kit and probes for RNA-scope were provided by Advanced Cell Diagnostics. Staining was assessed using the following scoring: quality: 0 = negative, 1 = single signal/cell, 2 = 2 to 4 signals/cell, 3 = ≥5 signals/cell; quantity: 0 = negative, 1 = <10% positive cells, 2 = 10 to 50% positive cells, 3 = >50 to 80% positive cells, 4 = >80% positive cells. Total score = quality × quantity: 0, absent; 1–4, weak; 5–8, moderate; 9–12, strong expression.

### 2.4. Cell Lines and Transfections

HepG2 cells (ATCC) were cultured in RPMI medium, while HuH7 (ATCC), HLE (JCRB), and HEK293T (ATCC) cell lines were grown in DMEM medium (Thermo Fisher Scientific, Waltham, MA, USA) at 37 °C (5% CO_2_). Culturing media were supplemented with 10% fetal bovine serum (Thermo Fisher Scientific, Waltham, MA, USA) and 1% penicillin-streptomycin (Thermo Fisher Scientific, Waltham, MA, USA). Authentication of cell lines was confirmed by STR profiling. All cell lines were tested routinely for mycoplasma contamination. Lentiviral particles were produced in HEK293T cells by combining the plasmid of interest with pMD2G and psPAX2 vectors (Addgene plasmids pMD2.G #12259 and psPAX2 #12260 both provided as a gift from Didier Trono). HLE cells were infected with lentiviral particles carrying either an empty or *LINC00152* cDNA-containing pLV-EF1a-IRES-Blast vector; pLV-EF1a-IRES-Blast was a gift from Tobias Meyer (Addgene plasmid #85133). HuH7^Δ^^*LINC00152*^ clones were generated by transfecting Cas9-positive HuH7 cells with a *LINC00152*-specific pair of single guide RNAs (sgRNA) subcloned in a pDECKO-mCherry plasmid (pDECKO-sg*LINC00152*) as previously described [19,20]; sgRNAs were designed using the CRISPETa tool (http://crispeta.crg.eu/, accessed on 1 July 2016) and cloned in the pDECKO backbone as described before [19,20]. Control cells were established following infection with lentiviral particles containing pDECKO-gGFP. pDECKO-mCherry and pDECKO-mCherry GFP plasmids were a gift from Roderic Guigo and Rory Johnson (Addgene plasmids #78534 and #78535, respectively). The sequences of all sgRNA are provided in the Table S4. Cas9-positive cells were established by infection with lentiviral particles carrying lentiCas9-Blast plasmid (Addgene plasmid #52962 as a gift from Feng Zhang). All siRNA transfections were carried out using Oligofectamine according to the manufacturer’s instructions (Thermo Fisher Scientific, Waltham, MA, USA); all siRNA sequences (Eurofins MWG Operon, Ebersberg, Germany) and final concentration used are also listed in Table S4. Cells were seeded in 6 well plates and harvested 72 h after siRNA transfection for RNA or protein isolation. For miRNA overexpression, cells were seeded in 6- or 96-well plates and were transfected with 10 pmol/µL miR-143a-3p (miRCURY LNA miRNA Mimic YM00470035-ADA from QIAGEN, Hilden, Germany) or control mimic (Eurofins) using Lipofectamine 2000 (Thermo Fisher Scientific, Waltham, MA, USA). For luciferase assay, HuH7 cells were transiently transfected in 6-well plates using Lipofectamine 2000 and 2.5 ng pRL-TK with 500 ng pMIR-*LINC00152*-Luc or pMIR-KLC2-Luc pMIR-empty plasmids [21] in combination with miR-143a-3p or control mimic; luciferase activity was quantified using the Dual-Glo Luciferase Assay System according to manufacturer’s instructions (Promega, Madison, WI, USA,) using a FLUOstar Omega Microplate Reader (BMG Labtech, Ortenberg, Germany).

### 2.5. Functional Analyses

A standard MTT assay (Methylthiazolyldiphenyl-tetrazolium bromide, Merck KGaA, Darmstadt, Germany) was used to determine the cell viability. Briefly, HepG2, HuH7, and HLE cells were plated at a density of 3 × 10^4^ cells/well in 96-well plates and treated as indicated in the respective figures. After adding DMSO/EtOH solution (1:2) to each well, colorimetric detection was carried out using a FLUOstar Omega Microplate Reader (BMG Labtech, Ortenberg, Germany). For colony formation assay, 4 × 10^3^ cells were seeded in 6-well plates and cell colonies were stained with PFA/crystal violet solution after 10 days. The colony intensity was determined by using the ImageJ software [22] and the relative clonogenicity was evaluated compared t, Germanyo the control treated cells. A two-dimensional scratch assay was performed as previously described [10]. In brief, 5 × 10^5^ HuH7 cells were plated in 6 well plates and transfected with siRNAs as described above. Two days after siRNA treatment, mitomycin C (5 μg/mL) was added to the cells for 3 h to inhibit cell proliferation before the cell monolayer was scratched by using a 200 µL pipette tip. Cells were then incubated with hepatocyte growth factor (HGF, 10 ng/mL; R&D Systems, Minneapolis, MN, USA) for 24 h to promote migration. The relative migratory capacity was determined using the ImageJ software as cell-covered area between the time points 0 and 24 h.

### 2.6. Total RNA, miRNA Isolation and qPCR

Total RNA from cell lines was isolated using the NucleoSpin RNA kit (Macherey-Nagel, Dueren, Germany) following the manufacturer’s instructions. CDNA synthesis was performed as reported previously [23]. MiRNAs were isolated from cells accordingly to miRNeasy kit instructions (QIAGEN, Hilden, Germany). The miQPCR approach was used to make cDNA synthesis as previously described [24]. Semi-quantitative real-time PCRs were performed using a QuantStudio3 device (Thermo Fisher Scientific, Waltham, MA, USA). The delta-delta CT method was used to quantify the relative gene expression compared to GAPDH or 18 s mRNA expression. Primer sequences are listed in Table S4.

### 2.7. Western Immunoblotting

For extraction of total proteins, cells were homogenized in lysis buffer (Cell Signaling, Frankfurt, Germany) after adding Protease Inhibitor Mix M (Serva, Heidelberg, Germany) and PhosStop Phosphatase Inhibitor Cocktail (Merck, Darmstadt, Germany). The NE-PER Nuclear and Cytoplasmic Extraction kit (Thermo Scientific, Offenbach, Germany) was used for nuclear and cytoplasmic fractionations, following the manufacturer’s protocol. Western immunoblotting was carried out as previously described [23]. Primary antibodies are listed in Table S5. IRDye-labeled secondary antibodies (Li-Cor, Li-Cor Biosciences, Lincoln, NE, USA) were incubated for 1 h at room temperature. Visualization of protein signal was carried out in an Odyssey CLx imaging device (Li-Cor, Lincoln, NE, USA). Densitometric analyses were performed by using Image Studio software (Li-Cor, Lincoln, NE, USA) and each specific protein band signal was normalized to the corresponding loading control, either ACTIN or GAPDH.

### 2.8. RNA Immunoprecipitation (RIP)

RIP was carried out according to the manufacturer’s instructions using the Magna RIP RNA-Binding Protein Immunoprecipitation Kit (Millipore, Merck KGaA, Darmstadt, Germany). In brief, HuH7 and HepG2 cells were plated in 15 cm dishes and collected at 80% confluency. RNA pull-down was performed by AGO2 protein immunoprecipitation. CDNA synthesis and real-time PCR were performed as described above. Relative *LINC00152* expression was calculated using the delta delta CT method compared to the IgG control following input normalization, while relative quantification of miRNA expression was performed following median normalization, using qBase software. Primer sequences are listed in Table S4.

### 2.9. Statistical Analysis

Statistical analyses were performed using Graph Pad Prism 8.02 (GraphPad Software, San Diego, CA, USA) and R software (http://www.R-project.org/, accessed on 12 May 2021). All data derived from at least three biological replicates are represented as mean with SD. For comparison of two groups, normality was assessed by the Shapiro-Wilk test. Data with normal distribution were analyzed by two-tailed or paired Student’s *t*-test with Welch’s correction when variances were unequal. The Wilcoxon test was used for the statistical analysis of KLC2, *LINC00152* and *miR-143a-3p* expression levels of LIHC samples. One-way ANOVA, followed by Tukey test, was used to compare more than two data groups. The association between *LINC00152* and *KLC2* expression in the human mRNA profiling data was calculated using Person’s R, while Spearman’s rho was applied to assess the semiquantitative expression data derived from *LINC00152* and KLC2 RNA in situ hybridization. For overall survival analysis, the Cutoff Finder application was used to define the optimal expression level cutting point to stratify HCC patients [25] from the TCGA cohort [4,17]. Univariate survival analysis was based on the Kaplan-Meier method, and a log-rank test was used for comparison of survival curves. A *p*-value < 0.05 was considered statistically significant.

## 3. Results

### 3.1. *LINC00152* Upregulation Promotes Tumorigenicity of Human Liver Cancer Cells

**Figure 1:**
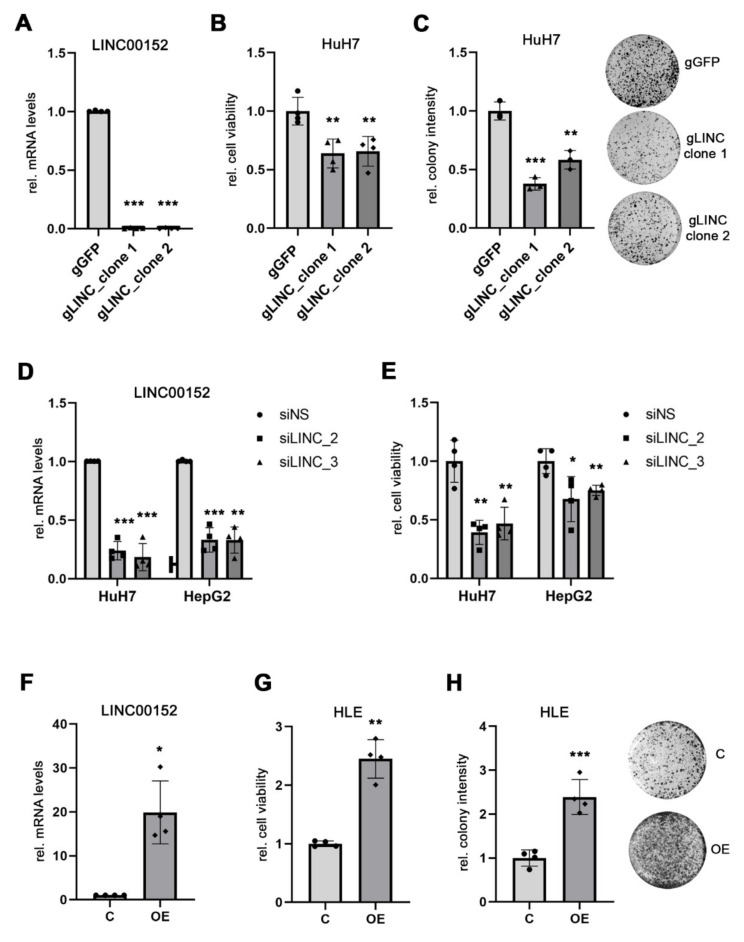
*LINC00152* expression affects cell viability of human HCC cell lines. (**A**) HuH7 cells 0 were engineered with CRISPR/Cas9 technology to achieve a complete *LINC00152* gene knock-out (HuH7^Δ*LINC00152*^). (**B**) Reduced cell viability of HuH7^Δ*LINC00152*^ clones compared to gGFP control cells. (**C**) Clonogenicity was significantly impaired in HuH7^Δ*LINC00152*^ clones compared to control cells. (**D**,**E**) Efficient siRNA-mediated *LINC00152* knockdown decreased cell viability of HuH7 and HepG2 cells. (**F**) Overexpression of *LINC00152* in HLE cells transfected with pLV-*LINC00152* plasmid. (**G**) *LINC00152* overexpression increased the cell viability of HLE cells compared to cells transfected with an empty vector. (**H**) Increased colony formation of *LINC00152*-overexpressing cells compared to control cells. Data are presented as mean ± SD of at least 3 independent experiments. Student *t*-test was used in (**A**–**C**,**F**), while Welch’s *t*-test in (**D**,**E**): * *p* < 0.05, ** *p* < 0.01, *** *p* < 0.001. Abbreviations: gGFP, guide RNA targeting GFP; gLINC, guide RNA targeting *LINC00152*; siNS, scrambled, nonsense siRNA; siLINC_2/_3, siRNA 2 and 3 specifically targeting *LINC00152*; C, control cells transfected with empty vector; *LINC00152* OE, *LINC00152* overexpression; OE, overexpression; rel., relative.

To characterize the function of *LINC00152* in human HCC cells, we generated *LINC00152* knockout cell lines (Δ*LINC00152*) using the DECKO system, a paired single guide RNAs CRISPR/Cas9 technology, specifically designed for the genomic perturbation of lnc-RNAs [19,20]. A complete knockout of *LINC00152* expression was achieved in HuH7 cells (Figure 1A). Both cell viability and colony formation capacity were significantly decreased in HuH7^Δ*LINC00152*^clones compared to unedited HuH7 control cells (Figure 1B,C). This finding was independently validated using gene-specific siRNA-mediated knockdown of *LINC00152* expression in HuH7 and HepG2 cells, respectively (Figure 1D,E). In addition, ectopic expression of *LINC00152* in low-expressing HLE cells promoted cell growth and clonogenicity compared to control cells transfected with an empty vector (Figure 1F–H).

### 3.2. *LINC00152* Is Enriched in RNP Complexes of Potentially *LINC00152* Binding miRNAs

In order to investigate whether *LINC00152* may constitute a pro-tumorigenic ceRNA network in human HCCs, we used an integrative strategy in which we combined in silico prediction of miRNA candidates and their target genes with a biostatistical approach to identify co-regulated genes. In silico, 22 miRNA families were predicted to bind to the *LINC00152* RNA sequence (Table S2). These were interrogated, together with their predicted 3796 mRNA target genes, leading to the identification of 14 miRNAs and 1230 mRNAs showing co-regulation in a cohort of human HCCs [10] and may thus constitute a *LINC00152*-driven ceRNA network. The top gene and miRNA candidates are shown in Table 1 and Table 2.

**Figure 2:**
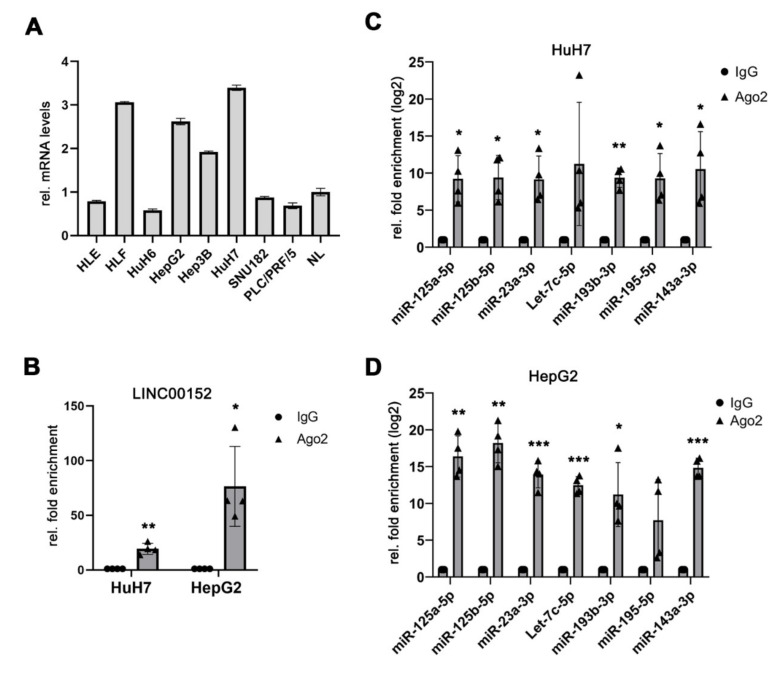
*LINC00152* and putative miRNAs were enriched in RNP complexes in a potential *LINC00152*-driven ceRNA network in human HCC. (**A**) *LINC00152* expression in human HCC cell lines. (**B**) *LINC00152* mRNA is enriched in AGO2 complexes of HCC cell lines compared to IgG control. (**C**,**D**) The predicted miRNAs components of the *LINC00152* ceRNA network are significantly more prevalent in AGO2-immunoprecipitated protein complexes of HuH7 and HepG2 cells compared to control. Data are presented as mean ± SD of 3 technical replicates in panel (**A**) or 4 independent experiments in (**B**–**D**). Welch’s *t*-test: * *p* < 0.05, ** *p* < 0.01, *** *p* < 0.001. Abbreviations: NL, normal liver tissue; AGO2, Argonaute-2; IgG, immunoglobulin G; rel., relative.

As miRNAs are part of ribonucleoprotein complexes (RNPs), which additionally contain RNA-binding proteins like Argonaute 2 (AGO2) [26,27], we performed an AGO2-RNA immunoprecipitation (IP) using HepG2 and HuH7 liver cancer cells, which showed high *LINC00152* expression levels (Figure 2A). *LINC00152* was significantly enriched in AGO2-RNA immuno-precipitates compared to the control immunoglobulin G (IgG) in both HCC cell lines, suggesting that it may be able to serve as a miRNA sponge (Figure 2B). In addition, five of the top 10 miRNA candidates of the predicted ceRNA network were also significantly more abundant in these AGO2-RNPs in both HuH7 and HepG2 cells compared to the control, respectively. Of note, miR-223-3p, miR-497-5p and miR-150-5p were not found enriched in theses RNP complexes and were thus not followed up further. Notably, Let-7c-5p and miR-195-5p were only detectable in one of the two cell lines analyzed and thus may act in a cell-type specific function (Figure 2C,D). In summary, *LINC00152* constitutes a component of RNPs in human liver cancer cell lines and may be able to regulate the bioavailability of the co-identified miRNAs via a *LINC00152*-driven ceRNA network.

### 3.3. Kinesin Light Chain 2 (KLC2) May Be a Component of a *LINC00152*-Driven ceRNA Network

**Figure 3:**
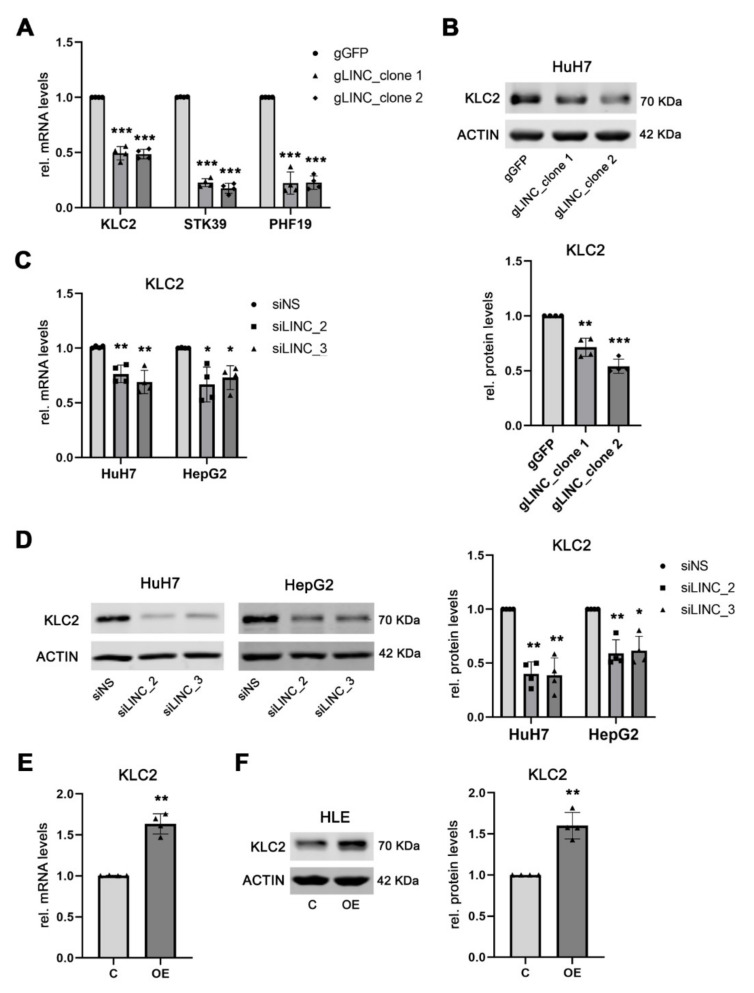
KLC2 belongs to the target genes of the *LINC00152*-driven ceRNA network. (**A**) *KLC2*, *STK39* and *PHF19* are significantly downregulated in HuH7^Δ*LINC00152*^ compared to HuH7 control cells. (**B**) Western blot analysis confirmed the decrease in KLC2 expression in HuH7^Δ*LINC00152*^ (**C**,**D**). Reduced KLC2 mRNA and protein levels were observed following *LINC00152* siRNA-medicated knockdown in HuH7 and HepG2 cells (**E**,**F**). Increased KLC2 mRNA and protein levels following overexpression of *LINC00152* in HLE cells. Data are presented as mean ± SD of 4 independent experiments. Welch’s *t*-test was used: * *p* < 0.05, ** *p* < 0.01, *** *p* < 0.001. Abbreviations: gGFP, guide RNA targeting GFP; gLINC, guide RNA targeting *LINC00152*; siNS, scrambled, nonsense siRNA; siLINC_2/_3, siRNA 2 and 3 specifically targeting *LINC00152*; C, control cells transfected with empty vector; *LINC00152* OE, *LINC00152* overexpression; OE, overexpression; rel., relative.

Next, we took advantage of HuH7^Δ*LINC00152*^ cells to validate the potential *LINC00152* target genes of the *LINC00152* ceRNA network in human HCC. In a functional ceRNA network, loss of the sponge increases the bioavailability of target miRNAs leading to reduced expression of their respective target genes. In line with this concept, the mRNA expression of Kinesin Light Chain 2 (*KLC2*), Serine/Threonine Kinase 39 (*STK39*), and PHD Finger Protein 19 (*PHF19*) were significantly reduced in HuH7^Δ*LINC00152*^ compared to control cells (Figure 3A), while other genes potentially involved in this network could not be validated, either indicating that these genes are not part of the *LINC00152* ceRNA network, or that these genes are not affected at the RNA levels (Figure S1). Moreover, a positive association between the mRNA expression levels of *LINC00152* with *KLC2*, *STK39*, and *PHF19* was detected in human HCC samples of the TCGAdataset (LIHC cohort [4,17]: R = 0.189, R = 0.301, R = 0.292, each *p* < 0.001, respectively), confirming that their expression could be regulated by a *LINC00152*-driven ceRNA network. While a pro-tumorigenic role of STK39 and PHF19 has already been proposed in human HCC [28,29], the potential function of KLC2 in liver cancer has remained elusive and was thus investigated in more detail. *LINC00152*-mediated regulation of KLC2 was also confirmed at the protein level (Figure 3B). Additionally, the dependency of the KLC2 expression level on the abundance of *LINC00152* was independently confirmed following siRNA-mediated knockdown of *LINC00152* expression in HepG2 and HuH7 cells, respectively (Figure 3C,D). Consistent with this finding, KLC2 was significantly upregulated, both at mRNA and protein levels, following ectopic *LINC00152* expression in HLE cells (Figure 3E,F).

### 3.4. *LINC00152* Controls the Bioavailability of miR-143a-3p

**Figure 4:**
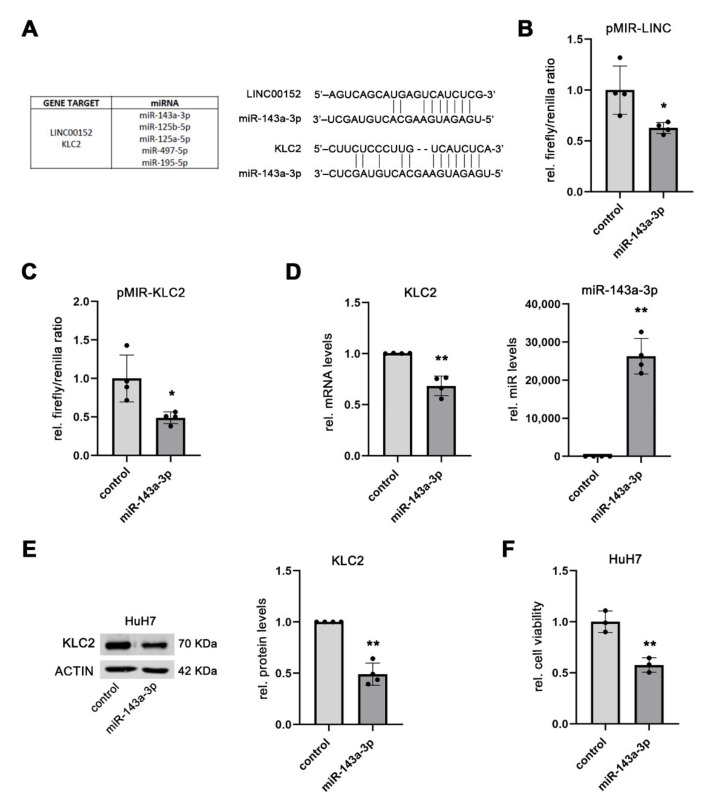
*LINC00152* controls the bioavailability of miR-143-3p. (**A**) Putative miRNA components of the *LINC00152* ceRNA network showing binding sites for both *LINC00152* and *KLC2*. (**B**,**C**) Reduced luciferase reporter activity due to binding of a miR-143a-3p mimic to *LINC00152* respectively *KLC2* in HuH7 cells. (**D**,**E**) Decreased KLC2 mRNA and protein levels following miR-143a-3p mimic transfection in HuH7 cells. (**F**) Transient miR-143a-3p overexpression lowered the cell viability of HuH7 compared to control cells. Data are presented as mean ± SD of at least 3 independent experiments. Student *t*-test in panel (**F**) and Welch’s *t*-test for (**A**–**E**): * *p* < 0.05, ** *p* < 0.01. Abbreviations: rel., relative.

Having shown a functional association between the expression levels of *LINC00152* and the predicted target genes of the proposed ceRNA network, we aimed to identify the miRNA that presumably regulates KLC2 expression. Based on the in-silico prediction, *KLC2* and *LINC00152* share miRNA binding sites for miR-143a-3p, miR-125a-5p, miR-125b-5p, miR-195-5p, and miR497-5p (Figure 4A). As miR-497-5p was not present in AGO2-RNP complexes in HepG2 and HuH7 cells and miR-195 and miR-125 had already been shown to bind to *LINC00152* in other cancer entities [30,31], miR-143a-3p was selected for further analysis. In the physical interaction between miR-143a-3p and *LINC00152*,, *KLC2* was confirmed using a dual-luciferase reporter assay, which showed that the luciferase activity, driven by either *LINC00152*- or a *KLC2*-specific nucleotide sequence, was significantly decreased following transfection of a miR-143a-3p mimic in HuH7 compared to control cells (1 ± 0.24 (mean ± SD; control) vs. 0.63 ± 0.05 (miR-143a-3p), *p* < 0.05 for pMIR-*LINC00152*; 1 ± 0.30 (control) vs. 0.49 ± 0.08 (miR-143a-3p), *p* < 0.05 for pMIR-KLC2) (Figure 4B,C). Furthermore, overexpression of miR-143a-3p in HuH7 cells significantly reduced KLC2 mRNA and protein levels compared to control cells (1 ± 0.002 (mean ± SD; control) vs. 0.68 ± 0.05 (miR-143a-3p), *p* < 0.01 for *KLC2* mRNA; 1 ± 0.00 (control) vs. 0.49 ± 0.11 (miR-143a-3p), *p* < 0.01 for KLC2 protein levels; Figure 4D,E), thus independently confirming its function as a regulator of KLC2. Of note, the cell viability of HuH7 cells, following overexpression of miR-143a-3p, was significantly reduced (1 ± 0.10 (mean ± SD; control) vs. 0.58 ± 0.07 (miR-143a-3p), *p* < 0.01; Figure 4F), suggesting a tumor suppressive role of miR-143a-3p in human HCC. All together, these data confirm that *LINC00152* is able to indirectly regulate *KLC2* expression by modulating miR-143a-3p bioavailability.

### 3.5. KLC2 Exerts Oncogenic Functions in Human HCC

**Figure 5:**
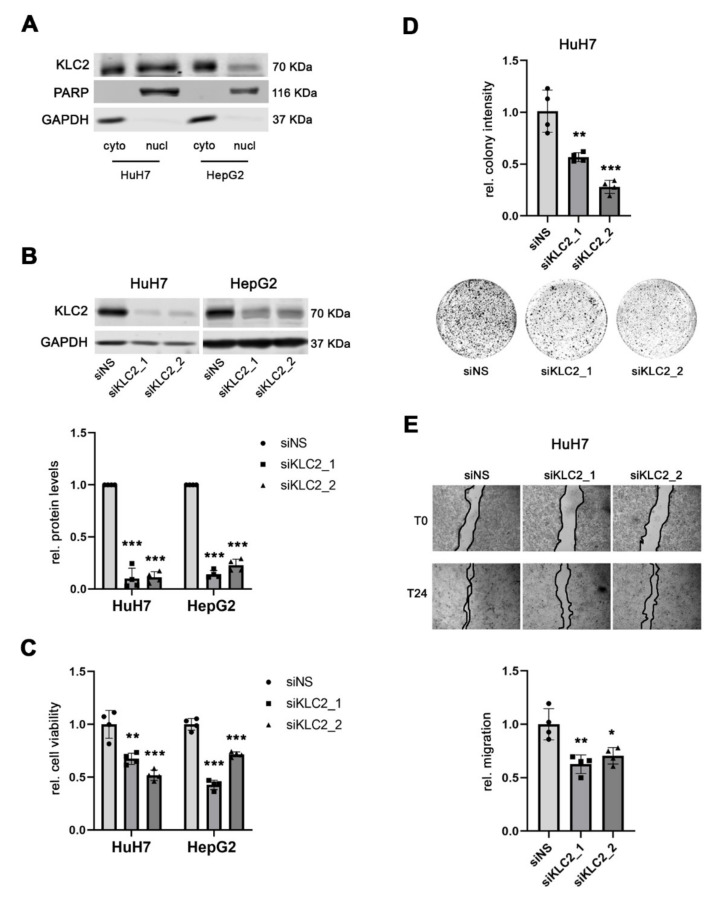
KLC2 exerts oncogenic functions in human HCC. (**A**) KLC2 is expressed both in the cytoplasmic and nuclear compartments of HuH7 and HepG2 cells. (**B**) Efficient siRNA-mediated knockdown of KLC2 protein levels in HuH7 and HepG2 cells compared to siNS control transfected cells. (**C**,**D**) Reduced cell viability and clonogenicity of both HuH7 and HepG2 cells following specific siRNA-mediated knockdown of KLC2 compared to siNS transfected control. (**E**) Migratory capacity of HuH7 cells either treated with KLC2-specific or siNS control siRNAs. Data are presented as mean ± SD of 4 independent experiments; in 5B only the upper error bar is displayed for graphical appearance reason. Welch’s *t*-test in (**B**), and unpaired *t*-test in (**C**–**E**): * *p* < 0.05, ** *p* < 0.01, *** *p* < 0.001. Abbreviations: cyto, cytoplasmatic protein fraction; nucl, nuclear protein fraction; siNS, scrambled, nonsense siRNA; siKLC2_1/_2, siRNA 1 and 2 specifically targeting KLC2; rel., relative.

Before investigating the function of KLC2 in human HCC, the cellular localization of the KLC2 protein was determined using fractionated protein isolates obtained from HepG2 and HuH7 cells, which showed both nuclear and cytoplasmic expression of the KLC2 protein (Figure 5A). Next, two independent gene-specific siRNAs were used to knockdown KLC2 in HuH7 and HepG2 cells, respectively (Figure 5B). At the functional level, siRNA-mediated inhibition of KLC2 expression significantly reduced the cell viability compared to the corresponding controls (1.00 ± 0.13 (mean ± SD; siNS) vs. 0.67 ± 0.05 (siKLC2_1) and 0.52 ± 0.05 (siKLC2_2), *p* < 0.01 and *p* < 0.001 in HuH7, respectively; 1.00 ± 0.06 (siNS) vs. 0.43 ± 0.04 (siKLC2_1) and 0.72 ± 0.02 (siKLC2_2), *p* < 0.001 in HepG2 cells; Figure 5C). Additionally, the ability to form colonies was significantly decreased following siRNA-mediated KLC2 knockdown in HuH7 compared to control cells (1.01 ± 0.20 (mean ± SD; siNS) vs. 0.57 ± 0.04 (siKLC2_1) and 0.28 ± 0.06 (siKLC2_2), *p* < 0.01 and *p* < 0.001 in HuH7, respectively; Figure 5D). Moreover, KLC2 knockdown impaired the migration of HuH7 compared to the control (1.00 ± 0.14 (mean ± SD; siNS) vs. 0.63 ± 0.09 (siKLC2_1) and 0.70 ± 0.08 (siKLC2_2), *p* < 0.01 for both siRNAs in HuH7; Figure 5E). Thus, KLC2 exerts pro-tumorigenic functions in liver cancer cells.

### 3.6. *LINC00152* and KLC2 RNAs Are Co-Expressed in Human HCC Cells

**Figure 6:**
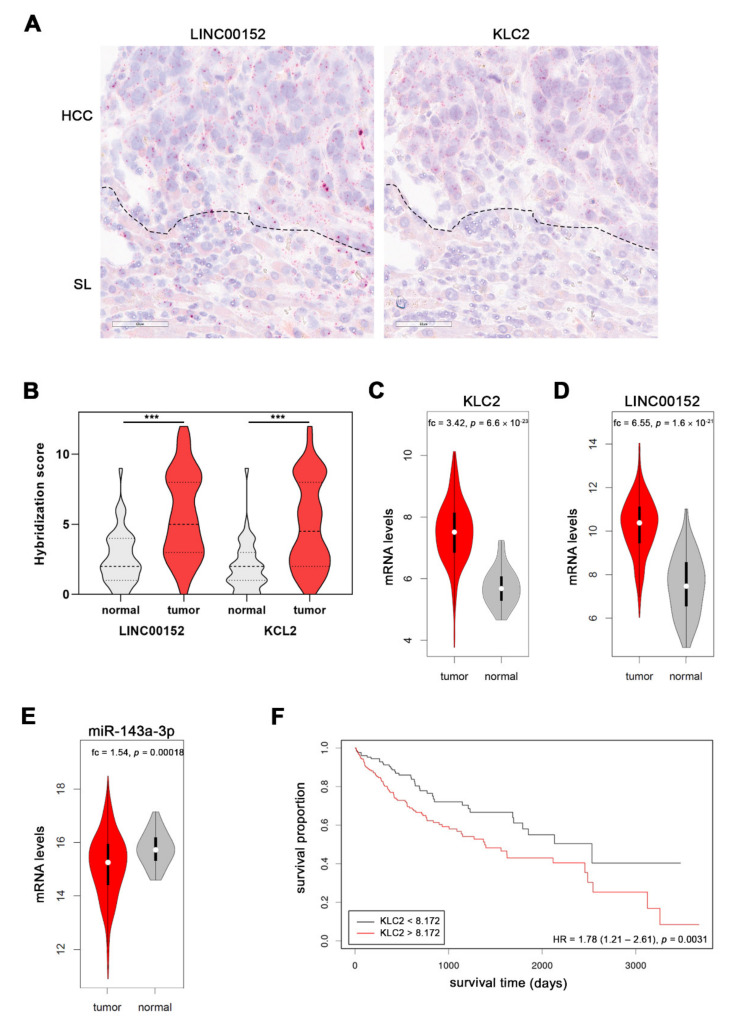
*KLC2* and *LINC00152* are co-expressed in HCC patients. (**A**) RNA hybridization signals of both *LINC00152*- and *KLC2*-specific RNA probes are more abundant in HCC cells compared to the surrounding non-tumorous liver (SL). Note, the co-expression of both transcripts. Dotted lines indicate the boundary between HCC and SL. Scale bar: 60 µM. (**B**) Semiquantitative scoring reveals upregulation of both *LINC00152* and *KLC2* transcripts in a human HCC cohort. (**C**,**D**) Upregulation of *KLC2* and *LINC00152* mRNA in human HCCs of the TCGA cohort. (**E**) Downregulation of miR-143a-3p expression in human liver tumors compared to normal tissues included in the TCGA dataset. (**F**) Kaplan-Meier curve showing shorter overall survival probability of HCC patients with higher *KLC2* expression levels (TCGA cohort). Paired *t*-test in panel B and Wilcoxon test in panel (**C**–**E**) *** *p* < 0.001. Abbreviations: SL, surrounding normal liver; fc, fold change.

To independently investigate whether *LINC00152* and *KLC2* RNA expression levels are associated in vivo, we took advantage of RNAscope technology and assessed their RNA levels in 50 human HCCs and corresponding non-tumorous surrounding liver (SL) tissues. We observed a significant increase of *LINC00152* RNA signals in human HCCs compared to SL (SL: 2.6 ± 0.3 (mean ± SEM) vs. 5.7 ± 0.4 (HCC); *p* < 0.001). This upregulation was paralleled by an upregulation of *KLC2* RNA signals (SL; 1.9 ± 0.3 (PT) vs. 4.9 ± 0.4 (HCC); *p* < 0.001; Figure 6A,B and Figure S2); as already shown in the TCGA dataset, a strong statistical association between *LINC00152* and *KLC2* RNA expression was observed (Spearman’s rho = 0.93, *p* < 0.001). Interestingly, *KLC2* and *LINC00152* were significantly upregulated in HCC compared to the surrounding non-tumor liver tissue in the TCGA dataset (LIHC cohort [4,17]: *KLC2* 5.73 ± 0.09 (mean ± SEM) (SL) vs. 7.51 ± 0.05 (HCC), *p* < 0.001, Figure 6C; *LINC00152* 7.51 ± 0.19 (mean ± SEM) (SL) vs. 10.22 ± 0.07 (HCC), *p* < 0.001, Figure 6D), while miR-143a-3p expression levels were significantly decreased in HCC compared to normal liver tissue in this cohort (15.76 ± 0.09 (mean ± SEM) (SL) vs. 15.14 ± 0.06 (HCC), *p* < 0.001, Figure 6E). Importantly, high *KLC2* expression was significantly associated with poor survival probability of HCC patients (Figure 6F). In addition, alpha-fetoprotein (AFP) serum levels showed a weak association with KLC2 expression (Spearman’s rho: 0.17, *p* = 0.047), which is in line with the well-known prognostic impact of high AFP expression in HCC patients. We did not observe an association between KLC2 expression and other clinical or pathological parameters (e.g., age, gender, performance status, progression-free or disease-specific survival, etiology, liver fibrosis stage, tumor grading, and vascular invasion; each *p* > 0.05).

In summary, *LINC00152* and *KLC2* are upregulated and co-expressed in liver cancer cells, providing further evidence that *KLC2* is a player in the *LINC00152*-driven ceRNA network in human HCC.

### 3.7. KLC2 Mediates the Oncogenic Functions of the *LINC00152* ceRNA Network in Liver Cancer Proliferation

**Figure 7:**
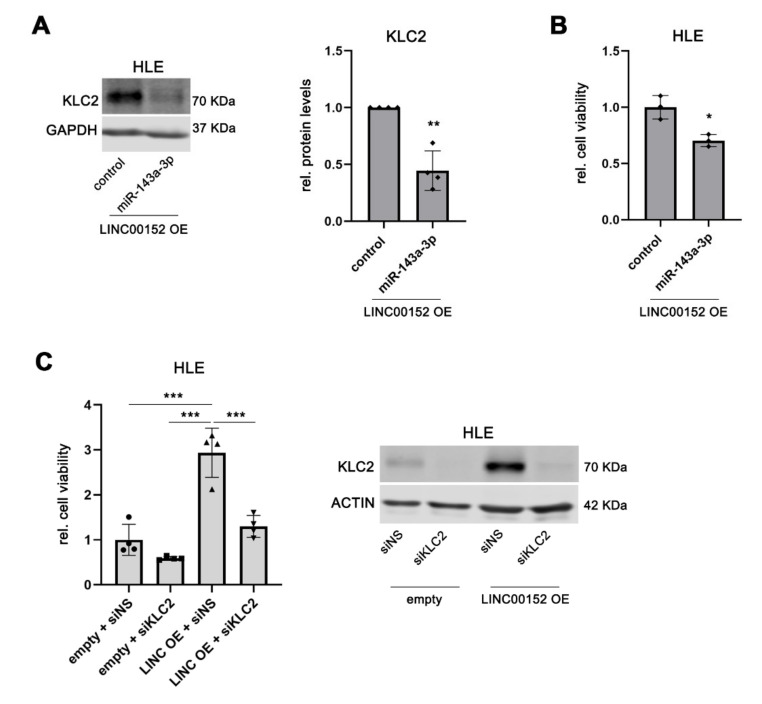
Mir-143a-3p mediates the KLC2 executed oncogenic phenotype of the *LINC00152* ceRNA network. (**A**) Reduced KLC2 protein levels following miR-143a-3p mimic transfection compared to control in *LINC00152* overexpressing cells. (**B**) MiR-143a-3p overexpression decreased cell viability of HLE cells overexpressing *LINC00152*. (**C**) KLC2 knockdown reduced cell viability of *LINC00152*-overexpressing HLE cells. Data are presented as mean ± SD of at least 3 biological replicates. Welch’s *t*-test in (**A**), unpaired *t*-test in (**B**) and one-way ANOVA followed by Tukey test in panel (**C**): * *p* < 0.05, ** *p* < 0.01, *** *p* < 0.001. Abbreviations: siNS, scrambled, nonsense siRNA; siKLC2, siRNA specifically targeting KLC2; *LINC00152* OE or LINC OE, *LINC00152* overexpression; OE, overexpression; empty, empty vector; rel., relative.

Finally, we aimed to confirm the functional impact of miR-143a-3p in regulating KLC2 within the *LINC00152* ceRNA network. For this purpose, *LINC00152*-overexpressing HLE cells, which showed increased KLC2 expression compared to control cells (Figure 3C,D), were transfected with a miR-143a-3p mimic. As shown in Figure 7A, KLC2 protein levels were significantly reduced following miR-143a-3p overexpression compared to *LINC00152*-overexpressing HLE control cells transfected with a control mimic (1.00 ± 0.0 (mean ± SD; control) vs. 0.45 ± 0.17 (miR-143a-3p), *p* < 0.01), confirming that *LINC00152* controls KLC2 expression via miR-143a-3p. As expected, miR-143a-3p overexpression significantly decreased the cell viability of *LINC00152*-overexpressing HLE cells (1.00 ± 0.10 (mean ± SD; control) vs. 0.70 ± 0.05 (miR-143a-3p), *p* < 0.05; Figure 7B), confirming that miR-143a-3p acts as a negative regulator of *KLC2* in the *LINC00152*-driven ceRNA network. In addition, the cell viability of *LINC00152*-overexpressing HLE cells was reduced following siRNA-mediated KLC2 knockdown compared to control siRNA transfected cells (Figure 7C). Of note, compared with siRNA-transfected HLE control cells the effect of *LINC00152* overexpression was completely rescued by siRNA-mediated knockdown of KLC2, suggesting that KLC2 is, indeed, the main pro-tumorigenic driver of the *LINC00152*-miR143a-3p-KLC2 axis (Figure 7C).

Overall, we characterized a complex epigenetic regulatory network, in which *LINC00152* regulates the bioavailability of miR-143a-3p via its sponging function, thereby counteracting the tumor suppressive function of miR-143a-3p exerted by inhibition of *KLC2* expression, which in turn promotes tumor growth of liver cancer cells.

## 4. Discussion

In recent years, lncRNAs have attracted enormous scientific attention due to their pivotal role in regulating crucial biological processes. As a result, dysregulation of lncRNAs has been linked to the pathogenesis of many diseases, including cancer. Although the function of many lncRNAs still remains elusive, or they have been incompletely characterized, several studies indicated that they are involved in chromatin organization and remodeling, as well as transcriptional, and post-transcriptional, regulatory processes [32]. One of the most intriguing features of lncRNAs is their ability to bind other RNA species, including miRNAs. Indeed, lncRNAs may act as a sponge in the sense that the abundance of a given lncRNA determines the bioavailability of the subset of miRNA species containing a homologous sequence, which, in turn, affects the expression level of the miRNA target genes within a cell; a constellation known as the ceRNA network [16]. Here, we identified, validated, and characterized a *LINC00152*-driven ceRNA network in human HCC. We not only defined the central mediators of this network, but also identified crucial effector genes mediating the pro-tumorigenic functions, in particular cell viability.

We have previously shown that hypomethylation of the *LINC00152* promoter results in overexpression of the gene in human HCC [10] and others have subsequently demonstrated an association between high levels of *LINC00152* expression with poor survival rate of HCC patients [33,34]. To our knowledge this is the first report of a convoluted epigenetic dysregulation in liver cancer, in which DNA hypomethylation of a lncRNA gene promoter leads to oncogenic activation of a ceRNA network.

Our in-silico approach allowed us to identify the potential miRNA constituents of the *LINC00152* miRNA network, most of which could be experimentally validated as components of *LINC00152* RNP complexes in human HCC cells. Thus, the *LINC00152*-miRNA interaction and the sponge function of *LINC00152* were confirmed. Furthermore, our findings demonstrate the high explorative power of our approach. Previous studies have described individual miRNAs binding to the *LINC00152* RNA sequence. Their binding was independently confirmed by us in liver cancer cells. In more detail, miR-125b-5p was described as a *LINC00152* binding partner in ovarian cancer and was shown to negatively regulate mitochondrial apoptosis signaling in this cancer entity [35]. Additionally, binding of miR-125a-5p to *LINC00152* was shown to increase the expression of the SRF gene by resulting in activation of Hippo and MAPK signaling pathways in human breast cancer [31]. Furthermore, *LINC00152* was shown to sequester miR-125b-5p, thereby promoting the expression of the miR-125b-5p target gene *KIAA1522*, and consequently the proliferation of human HCC cells [36]. Other studies showed the physical interaction of *LINC00152* with miR-193a-3p and miR-195 in several cancer entities, including HCC [30,37,38,39,40]. However, our study identified miR-23a and miR-143a-3p as new components of the *LINC00152* ceRNA network. Further investigations are needed to fully explore the role of miR-23a within the *LINC00152* network.

Using a *LINC00152* knockout approach *KLC2*, *PHF19*, and *STK39* were validated as being regulated by the *LINC00152* ceRNA network in liver cancer. PHF19, a member of the poly-comb group of proteins, which maintains the repressive transcriptional status of genes involved in development [41], was previously shown to be upregulated in human HCC and to promote tumor cell proliferation [29]. PHF19 is a known target gene of miR-195 [29], supporting the concept that the sponge function of *LINC00152* leads to an orchestrated activation of several oncogenic driver genes. Additionally, *LINC00152* has been reported to bind to Enhancer of the zeste homolog 2 (EZH2), a key component of the poly-comb repressor complex 2 (PRC2), in different cancer entities [42,43]. As we have previously reported, PCR2 target genes are prone to promote hypermethylation in human HCC [10]. It may be tempting to speculate that upregulation of *LINC00152* in cancer cells may even promote its own epigenetically-induced upregulation.

Recently, activation of the serine threonine kinaseSTK39 has been shown to promote HCC progression by activating PLK1/ERK signaling [28]; pathways frequently activated in human HCC [44,45]. In line with this, *LINC00152*-mediated activation of ERK/MAPK signaling has been described in gastric cancer [46].

Here, we provide the first evidence that upregulation of KLC2 promotes tumor growth in human cancer cells. KLC2 was previously known to function as a cargo adapter. In this function, it is able to form a hetero-tetrameric complex of the plus-end microtubule-motor protein Kinesin-1 with heavy chain KIF5 family members, a complex able to transport vesicles, protein complexes, and mRNAs [47]. KLC2 was shown to activate Smad2 signaling by mediating its transport along the microtubules to the axonal receptors in Xenopus and Zebrafish embryos [48]. Notably, overexpression of KLC2 was found in human HCC compared to non-neoplastic liver tissue in two independent cohorts, corroborating the previous observation that KLC2 is upregulated in non-small-cell lung cancer [49]. More importantly, higher KLC2 expression levels were associated with shorter overall survival of HCC patients. Of relevance, *KLC2* was co-expressed with *LINC00152* mRNA specifically in human HCC cells, demonstrating a tumor cell-specific mechanism, and independently validating the upregulation of both *KLC2* and *LINC00152* in an independent human HCC cohort. Mechanistically, sponged miR-143a-3p was shown as the mediator, promoting KCL2 overexpression in *LINC00152* overexpressing HCC cells. Overall, we constructed and validated a ceRNA network in which *LINC00152* sponges miR-143a-3p, thereby counteracting its negative regulation of KLC2 and thus promoting pro-tumorigenic KLC2 upregulation.

## 5. Limitations and Perspectives

Several lncRNAs have been reported to play pro-tumorigenic functions in human HCC [9], suggesting their enormous potential for the development of new therapeutic tests and innovative treatment approaches. While an anti-sense oligonucleotide- or siRNA-based approach showed promising results in HCC xenograft models [50,51], successful translation into clinical treatment of HCC patients has not yet been achieved. Therefore, a deeper understanding of the complexity of lncRNA-driven networks in HCC is fundamental for the development of new therapeutic approaches. Here, we dissected the *LINC00152*-driven ceRNA network in human HCC and identified miR-143a-3p and KLC2 as new players promoting tumor growth of human HCC. Due to the lack of a murine orthologue of the human *LINC00152* gene, the central limitation of our study is that further characterization, using a translationally relevant preclinical in vivo model, was not feasible. Nevertheless, our scientific strategy uncovered new potentially druggable targets mediating the oncogenic function of *LINC00152*, which can be addressed in suitable preclinical models to evaluate the translational potential of our findings.

## 6. Conclusions

KLC2 represents a key player of the *LINC00152*-driven ceRNA network in human HCC. Although more investigations are needed to fully explore the functions of KLC2 in liver cancer, targeting of central effector proteins may be an alternative option for personalized HCC treatment. In line with this, specific targeting of KLC2 was able to completely rescue the proliferative effect induced by *LINC00152* overexpression in vitro, suggesting the *LINC00152*-miR-143-KLC2 axis as a new potential therapeutic target in human HCC.

## Figures and Tables

**Table 1:** Top 20 candidate genes in *LINC00152*-driven ceRNA network.

Gene Symbol	Correlation Value *
*STK39*	0.59425
*FAM60A*	0.58052
*FUT4*	0.56763
*PALLD*	0.56334
*MAP3K1*	0.55911
*PLAU*	0.55639
*C15orf39*	0.54633
*ABCC5*	0.52205
*PODXL*	0.52193
*E2F3*	0.51697
*KLC2*	0.51462
*LRP12*	0.51185
*CHD7*	0.50999
*UBE2Q2*	0.50646
*PHF19*	0.50132
*FAM122B*	0.50077
*EIF2C2*	0.49204
*FAM60A*	0.48958
*LHFPL2*	0.48275
*RAP2B*	0.47997

* *p* value < 0.001.

**Table 2:** miRNA candidates within *LINC00152*-associated target network.

miRNA Symbol	*p* Value (Adjusted)
hsa-miR-23a-3p	0.000057
hsa-miR-125a-5p	0.000358
hsa-miR-125b-5p	0.000358
hsa-miR-223-3p	0.000401
hsa-miR-143-3p	0.001238
hsa-miR-497-5p	0.001238
hsa-let-7c	0.001870
hsa-miR-150-5p	0.002481
hsa-miR-195-5p	0.002895
hsa-miR-193b-3p	0.007043
hsa.let.7b.5p	0.011390
hsa-miR.18a.5p	0.021426
hsa-miR.139.5p	0.037874
hsa-let.7a.5p	0.049108

## Data Availability

Gene expression profiling data of human HCCs used in this study were previously deposited in GEO open access repository (GSE50579); TCGA data (LIHC cohort), were downloaded from the PanCanAtlas website (https://gdc.cancer.gov/about-data/publications/pancanatlas, accessed on 12 May 2021).

## Supplementary Materials

The following supporting information can be downloaded at: https://www.mdpi.com/article/10.3390/cells11091528/s1, Figure S1: Unchanged expression of some genes potentially involved in the *LINC00152*-driven ceRNA network; Figure S2: KLC2 and *LINC00152* RNAs are co-expressed in human HCC; Table S1: Patient’s characteristics of expression profiling cohort; Table S2: Predicted *LINC00152*-binding miRNAs; Table S3: Patient’s characteristics of TMA cohort; Table S4: Primer, siRNA and sgRNA sequence list; Table S5: Antibody list; Supplemental whole Western Blot Images.

## References

[1] Calderaro J., Couchy G., Imbeaud S., Amaddeo G., Letouze E., Blanc J.F., Laurent C., Hajji Y., Azoulay D., Bioulac-Sage P. (2017). Histological subtypes of hepatocellular carcinoma are related to gene mutations and molecular tumour classification. J. Hepatol..

[2] Shimada S., Mogushi K., Akiyama Y., Furuyama T., Watanabe S., Ogura T., Ogawa K., Ono H., Mitsunori Y., Ban D. (2019). Comprehensive molecular and immunological characterization of hepatocellular carcinoma. EBioMedicine.

[3] Montironi C., Castet F., Haber P.K., Pinyol R., Torres-Martin M., Torrens L., Mesropian A., Wang H., Puigvehi M., Maeda M. (2022). Inflamed and non-inflamed classes of HCC: A revised immunogenomic classification. Gut.

[4] Cancer Genome Atlas Research Network (2017). Comprehensive and integrative genomic characterization of hepatocellular carcinoma. Cell.

[5] Volders P.J., Anckaert J., Verheggen K., Nuytens J., Martens L., Mestdagh P., Vandesompele J. (2019). LNCipedia 5: Towards a reference set of human long non-coding RNAs. Nucleic Acids Res..

[6] Djebali S., Davis C.A., Merkel A., Dobin A., Lassmann T., Mortazavi A., Tanzer A., Lagarde J., Lin W., Schlesinger F. (2012). Landscape of transcription in human cells. Nature.

[7] DiStefano J.K., Gerhard G.S. (2022). Long noncoding RNAs and human liver disease. Annu. Rev. Pathol..

[8] Mahpour A., Mullen A.C. (2021). Our emerging understanding of the roles of long non-coding RNAs in normal liver function, disease, and malignancy. JHEP Rep..

[9] Huang Z., Zhou J.K., Peng Y., He W., Huang C. (2020). The role of long noncoding RNAs in hepatocellular carcinoma. Mol. Cancer.

[10] Neumann O., Kesselmeier M., Geffers R., Pellegrino R., Radlwimmer B., Hoffmann K., Ehemann V., Schemmer P., Schirmacher P., Lorenzo Bermejo J. (2012). Methylome analysis and integrative profiling of human HCCs identify novel protumorigenic factors. Hepatology.

[11] Yu Y., Yang J., Li Q., Xu B., Lian Y., Miao L. (2017). *LINC00152*: A pivotal oncogenic long non-coding RNA in human cancers. Cell Prolif..

[12] Ji J., Tang J., Deng L., Xie Y., Jiang R., Li G., Sun B. (2015). *LINC00152* promotes proliferation in hepatocellular carcinoma by targeting EpCAM via the mTOR signaling pathway. Oncotarget.

[13] Li S.Q., Chen Q., Qin H.X., Yu Y.Q., Weng J., Mo Q.R., Yin X.F., Lin Y., Liao W.J. (2020). Long intergenic nonprotein coding RNA 0152 promotes hepatocellular carcinoma progression by regulating phosphatidylinositol 3-Kinase/Akt/Mammalian target of rapamycin signaling pathway through miR-139/PIK3CA. Am. J. Pathol..

[14] Ma P., Wang H., Sun J., Liu H., Zheng C., Zhou X., Lu Z. (2018). *LINC00152* promotes cell cycle progression in hepatocellular carcinoma via miR-193a/b-3p/CCND1 axis. Cell Cycle.

[15] Xia T., Liao Q., Jiang X., Shao Y., Xiao B., Xi Y., Guo J. (2014). Long noncoding RNA associated-competing endogenous RNAs in gastric cancer. Sci. Rep..

[16] Salmena L., Poliseno L., Tay Y., Kats L., Pandolfi P.P. (2011). A ceRNA hypothesis: The Rosetta Stone of a hidden RNA language?. Cell.

[17] Liu J., Lichtenberg T., Hoadley K.A., Poisson L.M., Lazar A.J., Cherniack A.D., Kovatich A.J., Benz C.C., Levine D.A., Lee A.V. (2018). An integrated TCGA pan-cancer clinical data resource to drive high-quality survival outcome analytics. Cell.

[18] Longerich T., Breuhahn K., Odenthal M., Petmecky K., Schirmacher P. (2004). Factors of transforming growth factor beta signalling are co-regulated in human hepatocellular carcinoma. Virchows Arch..

[19] Aparicio-Prat E., Arnan C., Sala I., Bosch N., Guigo R., Johnson R. (2015). DECKO: Single-oligo, dual-CRISPR deletion of genomic elements including long non-coding RNAs. BMC Genom..

[20] Pulido-Quetglas C., Aparicio-Prat E., Arnan C., Polidori T., Hermoso T., Palumbo E., Ponomarenko J., Guigo R., Johnson R. (2017). Scalable design of paired CRISPR guide RNAs for genomic deletion. PLoS Comput. Biol..

[21] Castoldi M., Vujic Spasic M., Altamura S., Elmen J., Lindow M., Kiss J., Stolte J., Sparla R., D’Alessandro L.A., Klingmuller U. (2011). The liver-specific microRNA miR-122 controls systemic iron homeostasis in mice. J. Clin. Investig..

[22] Schneider C.A., Rasband W.S., Eliceiri K.W. (2012). NIH Image to ImageJ: 25 years of image analysis. Nat. Methods.

[23] Pellegrino R., Thavamani A., Calvisi D.F., Budczies J., Neumann A., Geffers R., Kroemer J., Greule D., Schirmacher P., Nordheim A. (2021). Serum Response Factor (SRF) drives the transcriptional upregulation of the MDM4 oncogene in HCC. Cancers.

[24] Benes V., Collier P., Kordes C., Stolte J., Rausch T., Muckentaler M.U., Haussinger D., Castoldi M. (2015). Identification of cytokine-induced modulation of microRNA expression and secretion as measured by a novel microRNA specific qPCR assay. Sci. Rep..

[25] Budczies J., Klauschen F., Sinn B.V., Gyorffy B., Schmitt W.D., Darb-Esfahani S., Denkert C. (2012). Cutoff Finder: A comprehensive and straightforward web application enabling rapid biomarker cutoff optimization. PLoS ONE.

[26] Izaurralde E. (2012). Elucidating the temporal order of silencing. EMBO Rep..

[27] Filipowicz W., Bhattacharyya S.N., Sonenberg N. (2008). Mechanisms of post-transcriptional regulation by microRNAs: Are the answers in sight?. Nat. Rev. Genet..

[28] Zhang C., Wang X., Fang D., Xu P., Mo X., Hu C., Abdelatty A., Wang M., Xu H., Sun Q. (2021). STK39 is a novel kinase contributing to the progression of hepatocellular carcinoma by the PLK1/ERK signaling pathway. Theranostics.

[29] Xu H., Hu Y.W., Zhao J.Y., Hu X.M., Li S.F., Wang Y.C., Gao J.J., Sha Y.H., Kang C.M., Lin L. (2015). MicroRNA-195–5p acts as an anti-oncogene by targeting PHF19 in hepatocellular carcinoma. Oncol. Rep..

[30] Zhang J., Li W. (2018). Long noncoding RNA CYTOR sponges miR-195 to modulate proliferation, migration, invasion and radiosensitivity in nonsmall cell lung cancer cells. Biosci. Rep..

[31] Liu Y., Li M., Yu H., Piao H. (2020). lncRNA CYTOR promotes tamoxifen resistance in breast cancer cells via sponging miR125a5p. Int. J. Mol. Med..

[32] Yao R.W., Wang Y., Chen L.L. (2019). Cellular functions of long noncoding RNAs. Nat. Cell Biol..

[33] Wang B., Yang S., Zhao W. (2020). Long non-coding RNA NRAD1 and *LINC00152* are highly expressed and associated with prognosis in patients with hepatocellular carcinoma. Onco. Targets Ther..

[34] Li J., Wang X., Tang J., Jiang R., Zhang W., Ji J., Sun B. (2015). HULC and *LINC00152* act as novel biomarkers in predicting diagnosis of hepatocellular carcinoma. Cell Physiol. Biochem..

[35] Chen P., Fang X., Xia B., Zhao Y., Li Q., Wu X. (2018). Long noncoding RNA *LINC00152* promotes cell proliferation through competitively binding endogenous miR-125b with MCL-1 by regulating mitochondrial apoptosis pathways in ovarian cancer. Cancer Med..

[36] Hu B., Yang X.B., Yang X., Sang X.T. (2020). LncRNA CYTOR affects the proliferation, cell cycle and apoptosis of hepatocellular carcinoma cells by regulating the miR-125b-5p/KIAA1522 axis. Aging.

[37] Li X., Rui B., Cao Y., Gong X., Li H. (2020). Long non-coding RNA *LINC00152* acts as a sponge of miRNA-193b-3p to promote tongue squamous cell carcinoma progression. Oncol. Lett..

[38] Liu P., He W., Lu Y., Wang Y. (2019). Long non-coding RNA *LINC00152* promotes tumorigenesis via sponging miR-193b-3p in osteosarcoma. Oncol. Lett..

[39] Wang H., Chen W., Yang P., Zhou J., Wang K., Tao Q. (2019). Knockdown of *LINC00152* inhibits the progression of gastric cancer by regulating microRNA-193b-3p/ETS1 axis. Cancer Biol. Ther..

[40] Huang Y., Luo H., Li F., Yang Y., Ou G., Ye X., Li N. (2018). *LINC00152* down-regulated miR-193a-3p to enhance MCL1 expression and promote gastric cancer cells proliferation. Biosci Rep..

[41] Ballare C., Lange M., Lapinaite A., Martin G.M., Morey L., Pascual G., Liefke R., Simon B., Shi Y., Gozani O. (2012). Phf19 links methylated Lys36 of histone H3 to regulation of Polycomb activity. Nat. Struct. Mol. Biol..

[42] Chen W.M., Huang M.D., Sun D.P., Kong R., Xu T.P., Xia R., Zhang E.B., Shu Y.Q. (2016). Long intergenic non-coding RNA 00152 promotes tumor cell cycle progression by binding to EZH2 and repressing p15 and p21 in gastric cancer. Oncotarget.

[43] Chen Q.N., Chen X., Chen Z.Y., Nie F.Q., Wei C.C., Ma H.W., Wan L., Yan S., Ren S.N., Wang Z.X. (2017). Long intergenic non-coding RNA 00152 promotes lung adenocarcinoma proliferation via interacting with EZH2 and repressing IL24 expression. Mol. Cancer.

[44] Pellegrino R., Calvisi D.F., Ladu S., Ehemann V., Staniscia T., Evert M., Dombrowski F., Schirmacher P., Longerich T. (2010). Oncogenic and tumor suppressive roles of polo-like kinases in human hepatocellular carcinoma. Hepatology.

[45] Dimri M., Satyanarayana A. (2020). Molecular signaling pathways and therapeutic targets in hepatocellular carcinoma. Cancers.

[46] Shi Y., Sun H. (2020). Down-regulation of lncRNA *LINC00152* suppresses gastric cancer cell migration and invasion through inhibition of the ERK/MAPK signaling pathway. OncoTargets Ther..

[47] Hirokawa N. (1998). Kinesin and dynein superfamily proteins and the mechanism of organelle transport. Science.

[48] Batut J., Howell M., Hill C.S. (2007). Kinesin-mediated transport of Smad2 is required for signaling in response to TGF-beta ligands. Dev. Cell.

[49] Wang M., Zhu X., Sha Z., Li N., Li D., Chen L. (2015). High expression of kinesin light chain-2, a novel target of miR-125b, is associated with poor clinical outcome of elderly non-small-cell lung cancer patients. Br. J. Cancer.

[50] Wang Y.L., Liu J.Y., Yang J.E., Yu X.M., Chen Z.L., Chen Y.J., Kuang M., Zhu Y., Zhuang S.M. (2019). Lnc-UCID promotes G1/S transition and hepatoma growth by preventing DHX9-mediated CDK6 down-regulation. Hepatology.

[51] Huang M., Wang H., Hu X., Cao X. (2019). lncRNA MALAT1 binds chromatin remodeling subunit BRG1 to epigenetically promote inflammation-related hepatocellular carcinoma progression. Oncoimmunology.

