# Supplementary material for: LINC00152 Drives a Competing Endogenous RNA Network in Human Hepatocellular Carcinoma
Source: Cells. 2022 May 3;11(9):1528. doi: 10.3390/cells11091528 (PMC9103153; doi:10.3390/cells11091528)
Supplement: Supplementary file 1 [file cells-11-01528-s001.zip › cells-1701855-supplementary.pdf]

Supplementary Materials

LINC00152 drives a competing endogenous RNA network in human hepatocellular carcinoma

Rossella Pellegrino [1,*], Mirco Castoldi [2], Fabio Ticconi [3], Britta Skawran [4], Jan Budczies.[1], Fabian Rose [1], Constantin Schwab [1], Kai Breuhahn [1], Ulf P. Neumann [5,6], Nadine T. Gaisa [7], Sven H. Loosen [2], Tom Luedde [2], Ivan G. Costa [3] and Thomas Longerich [1]

[1] Institute of Pathology, Heidelberg University Hospital, 69120 Heidelberg, Germany; jan.budczies@med.uni-heidelberg.de (J.B.); fabian.rose@med.uni-heidelberg.de (F.R.); constantin.schwab@med.uni-heidelberg.de (C.S.); kai.breuhahn@med.uni-heidelberg.de (K.B.); thomas.longerich@med.uni-heidelberg.de (T.L.)
[2] Clinic for Gastroenterology, Hepatology and Infectious Diseases, University Hospital Düsseldorf, Medical Faculty of Heinrich Heine University Düsseldorf, 40225 Düsseldorf, Germany; mirco.castoldi@med.uni-duesseldorf.de (M.C.); sven.loosen@med.uni-duesseldorf.de (S.H.L.); tom.luedde@med.uni-duesseldorf.de (T.L.)
[3] Institute for Computational Genomics, Joint Research Center for Computational Biomedicine, University Hospital RWTH Aachen, 52074 Aachen, Germany; fabio.ticconi@gmail.com (F.T.); ivan.costa@rwth-aachen.de (I.G.C.)
[4] Institute of Human Genetics, Hannover Medical School, 30625 Hannover, Germany; skawran.britta@mh-hannover.de
[5] Department of General, Visceral and Transplant Surgery, University Hospital RWTH Aachen, 52074 Aachen, Germany; uneumann@ukaachen.de
[6] Department of Surgery, Maastricht University Medical Centre, , 6229 HX Maastricht, The Netherlands
[7] Institute of Pathology, University Hospital RWTH Aachen, 52074 Aachen, Germany; ngaisa@ukaachen.de
* Correspondence: rossella.pellegrino@med.uni-heidelberg.de; Tel.: +49-(0)6221-56-34094.

Tables S1-S5

Figures S1-S2

Supplemental Whole Western Blot Figures

Table S1: Patient´s characteristics of expression profiling cohort

**Gender**		
	male	29 (76%)
	female	9 (24%)
**Etiology**		
	HBV	8 (21%)
	HCV	9 (24%)
	alcohol	7 (18%)
	cryptogenic	10 (26%)
	genetic hemochromatosis	3 (8%)
	alpha1-antitrypsin deficiency	1 (3%)
**Grading**		
	well differentiated HCC	5 (13%)
	moderately differentiated HCC	28 (74%)
	poorly differentiated HCC	5 (13%)
**Localization**		
	bilobar	2 (5%)
	left lobe	7 (18%)
	right lobe	19 (50%)
	unknown	10 (26%)
**Nodules (n=)**		
	single	24 (63%)
	up to three	5 (13%)
	more than three	9 (24%)
**Vascular invasion**		
	present	9 (24%)
**Metastasis**		
	intrahepatic	13 (34%)
	extrahepatic	2 (5%)
**UICC stage**		
	I	27 (71%)
	II	9 (24%)
	III	0 (0%)
	IV	2 (5%)
**Previous treatments**		
	liver resection	2 (5%)
	transarterial chemoembolisation	1 (3%)
**Liver fibrosis**		
	stage 1	5 (13%)
	stage 2	4 (11%)
	stage 3	5 (13%)
	stage 4	17 (45%)
	unknown	7 (18%)

Table S2. Predicted *LINC00152*-binding miRNAs

miRNA
hsa-let-7f-1-3p
hsa-miR-155-5p
hsa-miR-139-5p
hsa-miR-18a-5p
hsa-miR-139-3p
hsa-miR-125a-5p
hsa-miR-150
hsa-miR-143
hsa-miR-125a-3p
hsa-miR-193b
hsa-miR-223
hsa-let-7d-5p
hsa-miR-497
hsa-miR-195
hsa-miR-23a
hsa-miR-125b
hsa-let-7g
hsa-let-7c
hsa-let-7a
hsa-let-7b-5p
hsa-miR-216a-5p
hsa-miR-138-5p

Table S3: Patient´s characteristics of TMA cohort

**Gender**		
	male	39 (78%)
	female	11 (22%)
**Etiology**		
	HBV	9 (18%)
	HCV	5 (10%)
	co-infection	1 (2%)
	alcohol	3 (6%)
	metabolic syndrome	5 (10%)
	alcohol + metabolic syndrome	3 (6%)
	primary biliary cholangitis	1 (2%)
	unknown	23 (46%)
**Grading**		
	well differentiated HCC	6 (12%)
	moderately differentiated HCC	22 (44%)
	poorly differentiated HCC	22 (44%)
**UICC stage**		
	I	16 (32%)
	II	21 (42%)
	III	12 (24%)
	IV	1 (2%)
**Vascular invasion**		
	present	22 (44%)
**Liver cirrhosis**		
	present	25 (50%)

Table S4: Primer, siRNA and sgRNA sequence list

Gene name	Sequence
GAPDH-fw	5′-TGCACCAACTGCTTAGC-3′
GAPDH-rev	5′-GGCATGGACTGTGGTCATGAG-3′
18s-fw	5′-AAACGGCTACCACATCCAAG-3′
18s-rev	5′-CCTCCAATGGATCCTCGTTA-3′
LINC00152-fw	5′-CCACCAGCCTCTCCTTGAATA-3′
LINC00152-rev	5′-GGCTGAGTCGTGATTTTCGG-3′
FUT4-fw	5′-GGTCCGCTACTACCACCAAC-3′
FUT4-rev	5′-CGAGTTCTCGAAAGCCAGGT-3′
STK39-fw	5′-CAGGAGGTTATCGGCAGTGG-3′
STK39-rev	5′-CACGTTCTTGCCTGGGTTTG-3′
MAP3K1-fw	5′-GCAGCGTTCTGTCAATGGTC-3′
MAP3K1-rev	5′-ACCAGCATGGCTCTCAATGT-3′
ABCC5-fw	5′-AGCAGGGGCGCAGGAAT-3′
ABCC5-rev	5′-GTGCTGGTTCTCTCCCTCAC-3′
E2F3-fw	5′-GGAGCTAGGAGAAAGCGGTC-3′
E2F3-rev	5′-TGAGGGAGATTTTGGAGTTTTTGG-3′
KLC2-fw	5′-AGGGGATGTGTCTGGTCAG-3′
KLC2-rev	5′-CCTGTGAGGCGTATTGGATCA-3′
PLAU-fw	5′-CGCAGCCACCGAGCC-3′
PLAU-rev	5′-CTTTGGAGTCGCTCACGACC-3′
UBE2Q2-fw	5′-GTCAGTTGGAAGCTGGACGA-3′
UBE2Q2-rev	5′-AGGATTCCGTGATGTTGCAGT-3′
PHF19-fw	5′-ACTGGCTGTGCGGAAAGG-3′
PHF19-rev	5′- AGGACAGCACCATCTTCACG -3′
hsa-let-7c-5p	5′-GTAGTAGGTTGTATGGTTG-3′
hsa-miR-125a-5p	5′-AGACCCTTTAACCTGTGAG-3′
hsa-miR-125b-5p	5′-GAGACCCTAACTTGTGAG-3′
hsa-miR-143-3p	5′-ATGAAGCACTGTAGCTCG-3′
hsa-miR-155-5p	5′-CTAATCGTGATAGGGGTTG-3′
hsa-miR-193b-3p	5′-CCCTCAAAGTCCCGCTG-3′
hsa-miR-195-5p	5′-CAGCACAGAAATATTGGCG-3′
hsa-miR-23a-3p	5′-CATTGCCAGGGATTTCCG-3′
mQRT	5′-CCCAGTTATGGCCGTTTATGCAGGT-3′
UPm2A	5′-CCCAGTTATGGCCGTTTA-3′
RNU6	5′-GCAAGGATGACACGCAAATT-3′
siKLC2_1 (final concentration 40nM)	5′-AGGACAGCACCATCTTCACG-dTdT-3′
siKLC2_2 (final concentration 40nM)	5′-CTGGTACAAGGCCTGTAAA-dTdT-3′
siLINC00152_2 (final concentration 60nM)	5′-GGAGATGAAACAGGAAGCT-dTdT-3′
siLINC00152_3 (final concentration 60nM)	5′-TCTATGTGTCTTAATCCCTTGTCCT-dTdT-3′
nonsense siRNA (siNS)	5′-TTCTCCGAACGTGTCACGT-dTdT-3′

sgLINC00152_1	5′-GCAGCCTCAGAAATACAAAA-3′
sgLINC00152_2	5′-CGGGATATCGGGTGGCGGCT-3′

Table S5: Antibody list

Gene name	Species	Catalog Number	Vendor
KLC2	Rabbit Polyclonal	ab254848	Abcam (Cambridge, MA USA)
GAPDH	Mouse Monoclonal	MCA4739	Bio-Rad Laboratories (Hercules, CA, USA)
PARP	Rabbit Polyclonal	#9542	Cell Signaling (Danvers, MA, USA)
β-ACTIN	Mouse Monoclonal	A5441	Sigma-Aldrich (St. Louis, MO, USA)
Ago2	Rabbit Monoclonal	03-110	Millipore, Merck (Darmstadt, Germany)

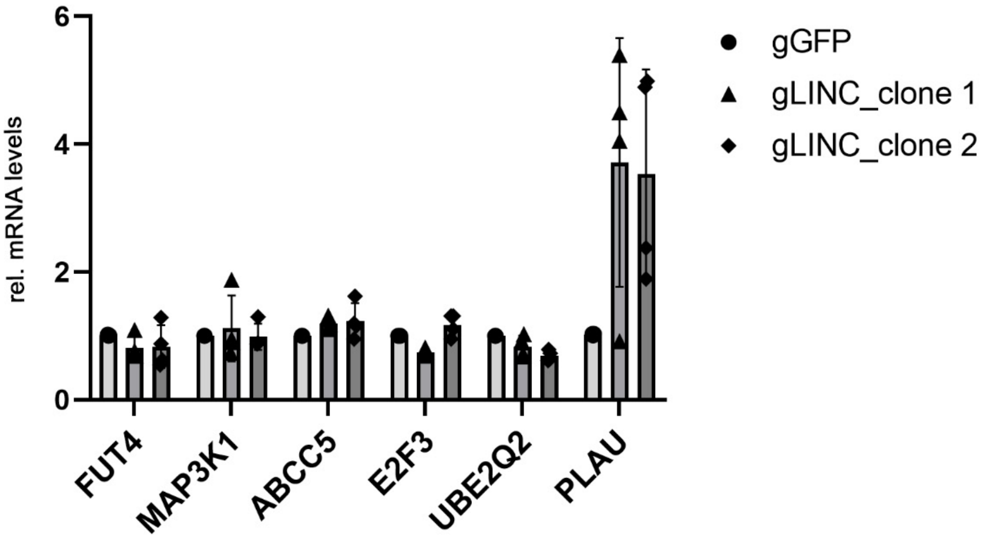

Figure S1. Unchanged expression of some genes potentially involved in the LINC00152-driven ceRNA network. Data are represented as mean ± SD of 4 biological replicates. Abbreviations: gGFP, guide RNA targeting GFP; gLINC, guide RNA targeting *LINC00152*; rel., relative.

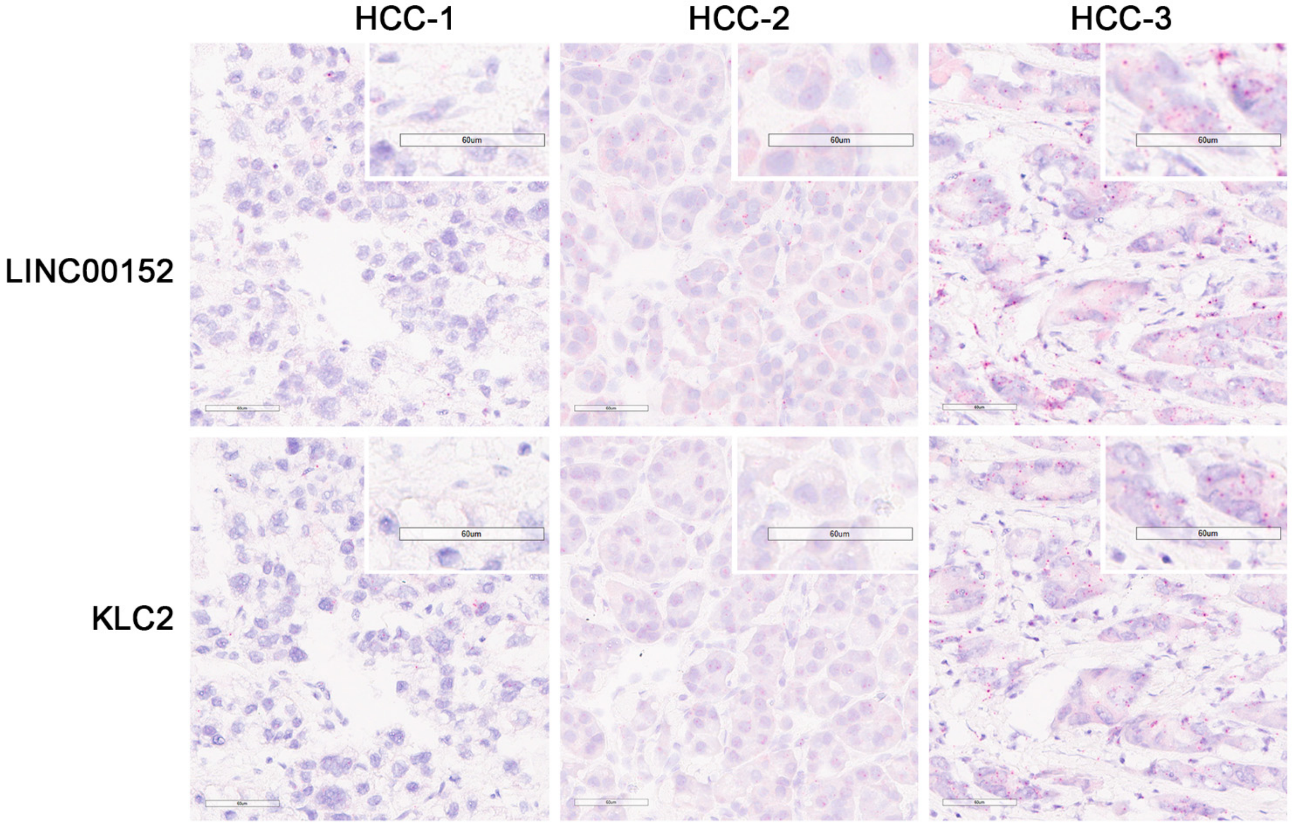

Figure S2. KLC2 and LINC00152 RNAs are co-expressed in human HCC. Additional representative images of human HCCs from TMA analysed by RNA-scope technology in Figure 5B; high LINC00152 RNA signals are associated with high KLC2 levels and vice versa.
